# Percutaneous Cryoablation of Pulmonary Metastases from Colorectal Cancer

**DOI:** 10.1371/journal.pone.0027086

**Published:** 2011-11-09

**Authors:** Yoshikane Yamauchi, Yotaro Izumi, Masafumi Kawamura, Seishi Nakatsuka, Hideki Yashiro, Norimasa Tsukada, Masanori Inoue, Keisuke Asakura, Hiroaki Nomori

**Affiliations:** 1 Department of Surgery, School of Medicine, Keio University, Tokyo, Japan; 2 Department of Radiology, School of Medicine, Keio University, Tokyo, Japan; 3 Department of Surgery, School of Medicine, Teikyo University, Tokyo, Japan; Emory University, United States of America

## Abstract

**Objective:**

To evaluate the safety and efficacy of cryoablation for metastatic lung tumors from colorectal cancer.

**Methods:**

The procedures were performed on 24 patients (36–82 years of age, with a median age of 62; 17 male patients, 7 female patients) for 55 metastatic tumors in the lung, during 30 sessions. The procedural safety, local progression free interval, and overall survival were assessed by follow-up computed tomographic scanning performed every 3–4 months.

**Results:**

The major complications were pneumothorax, 19 sessions (63%), pleural effusion, 21 sessions (70%), transient and self-limiting hemoptysis, 13 sessions (43%) and tract seeding, 1 session (3%). The 1- and 3-year local progression free intervals were 90.8% and 59%, respectively. The 3-years local progression free intervals of tumors ≤15 mm in diameter was 79.8% and that of tumors >15 mm was 28.6% (p = 0.001; log-rank test). The 1- and 3-year overall survival rates were 91% and 59.6%, respectively.

**Conclusion:**

The results indicated that percutaneous cryoablation is a feasible treatment option. The local progression free interval was satisfactory at least for tumors that were ≤15 mm in diameter.

## Introduction

Pulmonary metastases are common in patients with colorectal cancer. The 5-year probability of survival was found to be 27–40% [1], [2], [3], [4], [5], [6], [7] in patients who had undergone surgical resection. In 1965, Thomford et al., reported criteria for resection of metastatic lung tumors which have been generally accepted by the surgical community [8]. Resectability depends on the number and location of lesions, patient's age, comorbidities and pulmonary function tests as well as the ECOG performance status. Nonsurgical candidates are usually managed with systemic chemotherapy. As for chemotherapy, newer systemic chemotherapeutic regimens for metastatic colorectal cancer have recently shown improved response rates (35–50%) and overall survival (18–20months) [9], [10], [11]. Still, a less invasive local therapeutic option is desirable, that is as effective as surgery.

Many interventional radiologists regard cryoablation as an intraoperative modality used by urologists and general surgeons for prostate gland [12] and liver tumor ablations [13], respectively. Although percutaneous cryoablation of the thorax has also been reported in 2005 [14], percutaneous cryoablation is still not as widely applied as radiofrequency ablation for lung tumors [15]. During the cryoablation, the cryoprobe uses high-pressure argon and helium gases for freezing and thawing, respectively, on the basis of the Joule-Thompson principle. The ice ball can be detected by CT as ground glass opacity. The air in the lung can interfere with the creation of the ice ball. When the cryoprobe is inserted into normal pulmonary parenchyma, initial freezing causes the formation of an ice ball with a diameter of only 1 cm because the air prevents conduction of low temperatures, and there is not enough water in the parenchyma. However, after thawing, the massive intra-alveolar hemorrhage excludes the air and results in the formation of a larger ice ball in the subsequent freezing step. We therefore performed 3 freeze/thaw cycles to freeze a volume of tissue 2.5–3.0 cm in diameter.

We have previously reported the preliminary results of percutaneous cryoablation for lung tumors performed under computed tomography (CT) guidance with local anesthesia as a local curative treatment, which showed promising perioperative outcomes and local disease control in a mixed group of tumors [16]. However, the survival benefits of cryoablation remain unclear, and the type of patients that would benefit from this procedure is also unknown. In order to evaluate the safety and efficacy of this procedure for pulmonary metastasis from colorectal cancer, we have retrospectively reviewed the safety, the local progression free interval, and the survival rates in 24 nonsurgical candidates in this study. To our knowledge, this is the first report of the feasibility as well as the local control potential of cryoablation which focuses specifically on lung metastasis from colorectal cancer, which is one of the most frequently encountered metastatic lesions in the lung.

## Methods

### Ethics

This study protocol was approved by Keio University institutional review board (approval ID: 14–23). Written informed consent was obtained from each participant in accordance with the Declaration of Helsinki.

### Patient Selection

This was a retrospective study of patients treated for metastatic pulmonary tumors from colorectal cancer in our institution from November 2002 through May 2007. Patients with more than 3 years of follow-up were reviewed.

A patient was deemed non surgical due to any of the following: (1) multiple previous thoracotomies (2) multifocal disease (3) concomitant tumors (4) patient refused surgery, (5) respiratory dysfunction, or (6) advanced age. The threshold of respiratory dysfunction was below 800 mL with predicted post-operative forced expiratory volume in one second. Written informed consent was obtained from all patients. The exclusion criteria were as follows: (1) The ECOG performance status was 2 or 3. (2) The platelet count was less than 50,000/µL. (3) The prothrombin time international normalized ratio was more than 1.5. (4) The largest tumor diameter was greater than 40 mm as measured by preoperative volumetric CT. (5) There was no suitable way for the insertion of probes due to interference by major vasculatures, airways or mediastinal structures. (6) The primary colorectal carcinoma had not been controlled. (7) The patient was deemed incapable of cooperating during the procedure.

When the patient had metastases in bilateral lungs, we performed the treatment sequentially on different days.

### Cryoablation Technique

All the cryoablation procedures were performed using a multidetector CT scanner (Aquilion 64; Toshiba Med. Co. Ltd., Tokyo, Japan), which enables use of multislice CT fluoroscopy. Before leaving the ward, each patient received an intramuscular injection of atropine sulfate (0.5 mg) and pentazocine (15 mg), used for the premedication to ally patients' anxiety and reduce the bowel movement during the procedure. Cryoablation was performed after administration of local anesthesia by a thoracic surgeon or by a radiologist. A twenty-one-gauge guide needle was inserted into the targeted tumor under CT-fluoroscopic guidance. The needle always penetrated the tumor and the tip of the needle was placed at the far end of the lesion. With the needle in optimal position, a coaxial stainless steel introducer for the cryoprobe was inserted over the needle. The introducer consisted of inner tapered obturator and external sheath. The inner and outer diameters of the external sheath for a 1.7-mm-diameter cryoprobe (CRYOcare Cryosurgical Unit; Endocare, Irvine, CA) were 2 and 3 mm, respectively. The area of the heat exchanger segment is 4 cm from the tip of both types of the probes. After the guiding needle and inner sheath were removed, a cryoprobe was inserted through the external sheath. The sheath was 180 mm long, equal to the length of the cryoprobe, and therefore the cryoprobe tip was located at the end of the sheath. Cryoablation procedure consisted of 3 freezing cycles (one cycle of 5 minutes and 2 cycles of 10 minutes) separated by 3 thawing cycles during which the probe was allowed to reach a temperature of 20°C. There is a thermocouples device built into the cryoprobe. The probe temperature was monitored real-time automatically. The 1.7 mm-diameter cryoprobes can freeze a volume of tissue with a diameter of 2 cm [17], [18], and measuring 4 cm in length, after 3 cycles of freezing and thawing. Therefore, for tumors smaller than 2 cm, only 1 cryoprobe is usually inserted, and for 2- to 3-cm tumors, 2 or more cryoprobes are used to freeze a more-than-3-mm thick rind of normal tissue around the tumor. It takes 20–30 min to place a guide needle into the optimal position in the targeted tumor, and cryoablation starts simultaneously after all cryoprobes are placed. Once cryoablation is completed, fibrin glue (Bolheal®, The Chemo-Sero-Therapeutic Research Institute, Kumamoto, Japan) is injected through the sheath to reduce the risk of bleeding and pneumothorax. One ml of fibrinogen and 1 ml of thrombin per one probe were injected into the sheath simultaneously.

### Evaluation and Statistical Analysis

Complications were recorded and classified in accordance with the Common Terminology Criteria of Adverse Events (CTCAE) v4.0. We also evaluated radiographic local tumor control and overall survival. Radiographic local tumor control was assessed by criteria published in 2005 [19]. A post-operative plain thoracic CT was performed immediately after the removal of the cryoprobes for verification of any major complications such as air embolism, hemothorax, or tension pneumothorax, had occurred. CT scans were also performed at both 1 day and 1 week after the cryoablation procedure. Follow-up dynamic CT chest scans of patients were carried out at 1-month and then at 3- to 4-month intervals. Unenhanced chest CT was performed in patients with iodine allergies. The modified RECIST criteria [20] were basically used to assess response. CT scans in every case are reviewed by three diagnostic radiologists (M.I., H.Y. and S.N.) with 13, 14 and 20 years of clinical experiences, respectively, to determine whether progression had occurred. Diagnoses were made independently, and these radiologists discussed when they turned out to be different. In some cases, consolidation with ground grass opacity located adjacent to the treated lesion makes it difficult to detect the tumor progression. In such cases, a CT-guided needle biopsy was also performed to obtain a definitive diagnosis. Kaplan-Meier with log-rank analysis was used to analyze local progression free interval and cumulative survival after the initial cryoablation. All statistical analyses were performed using SPSS 17.0 software (SPSS Inc., Chicago, IL, USA). Statistical significance was set at p<0.05.

## Results

### Clinical Data

Percutanous cryoablation was performed on 24 patients (36–82 years of age, median age: 62 years; 17 male patients, 7 female patients) for 55 metastatic lung tumors from colorectal cancer in 30 sessions. Most of the patients had undergone treatment for lung metastases before cryoablation: 17 patients had received chemotherapy, 14 patients had undergone lung surgery, and 2 patients had undergone stereotactic radiosurgery. Therapeutic option of cryoablation was chosen according to the criteria stated earlier. Three patients refused surgery; twenty-one patients were nonsurgical candidates due to multiple tumors or insufficient pulmonary function (Table 1). The cryoablation procedures were well-tolerated by all patients under local anesthesia. Two patients required an additional injection of pentazocine (15 mg) during the procedure. The mean procedure time was 136±37 min (range, 66–192 min).

**Table 1:** Patient characteristics.

	Number of patients
Sex	
Male	17
Female	7
Age (yr)	
Average	62.0
Range	36–82
Number of lesions treated	
1	11
2	7
3 or more	6
Past history of other metastasis	
Lung	14
Liver	5
Brain	1
Prostate	1
Reasons for cryoablation	
Refusal of surgery	3
Unresectability	21
Multiple metastases	12
Insufficient pulmonary function	12
Old age	1
Treatment for pulmonary metastasis before cryoablation	
Chemotherapy	17
Surgery	14
Single surgery	7
More than 2 surgeries	7
Radiation	2

The mean tumor diameter was 13±7 mm (range, 3–31 mm). Of the 55 lesions, 34 lesions were treated by using 1 cryoprobe; 19 lesions, by 2 cryoprobes; and 2 lesions, by 3 or more cryoprobes (Table 2). Three tumors were too hard to be transfixed by the probe. For these tumors, two probes were placed adjacent to the tumors.

**Table 2:** Characteristics of tumors.

	Number of tumors
Tumor diameter (mm)	
Average	13
Range	3–31
Local control	
Local recurrence	10
No recurrence	45
Number of cryoprobes per tumors	
1	34
2	19
>3	2

### Perioperative Outcomes

Pneumothorax occurred in 19 sessions (63%) with 16 patients (66%), mostly immediately after the completion of the ablation procedure. The insertion of a chest tube was required in 1 session (3%) in one patient (4%). A small amount of pleural effusion occurred in 21 sessions (70%) in 17 patients (71%), but none of the patients required the insertion of a chest tube to control the effusion. Transient hemoptysis occurred after cryoablation in 13 sessions (43%) in 10 patients (42%), and during cryoablation in 3 sessions (10%) in 3 patients (12.5%). No interventions were required in any of these patients. In all cases, perioperative pain was controlled by loxoprofen, which was stopped within 1 week. Some patients in our study complained of dull pain in the anterior chest soon after treatment, which was probably due to intercostal nerves' damage. However, their pain usually resolved within a few months. There were neither treatment-related deaths nor conversions to surgical intervention. A tract seeding was observed in 1 case, 5 months after cryoablation. The tumor was controlled by surgical resection of the chest muscles and skin.

### Local progression free interval and overall survival

**Figure 1:**
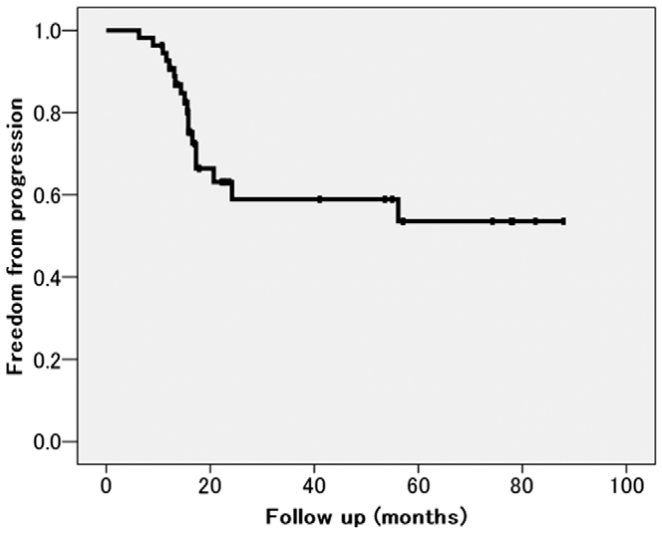
Local progression free interval curve. The median follow-up period was 40 months. Up to the date of the last follow-up of each patient, 17 tumors (26%) showed disease progression at the original cryoablation site, and hence median local progression free interval could not be determined. Local progression free interval at 1- and 3-years after treatment was 90.8% and 59%, respectively.

The median follow-up period was 40 months. Up to the date of the last follow-up of each patient, 17 tumors (26%) showed disease progression at the original cryoablation site, and hence median local progression free interval could not be determined. Local progression free interval at 1- and 3-years after treatment was 90.8% and 59%, respectively (Figure 1).

**Figure 2:**
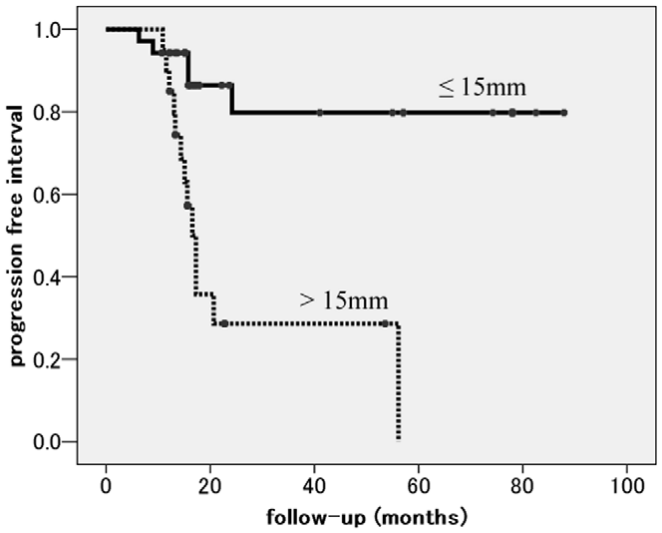
Local progression free interval curve according to tumor diameter. Local progression free interval was significantly greater for lesions with a diameter less than 15 mm. The 3-years local progression free interval of tumors ≤15 mm in diameter was 79.8% and that of tumors >15 mm in diameter was 28.6% (p = 0.001, log-rank test).

Local progression free interval was significantly greater for lesions with a diameter less than 15 mm. The 3-years local progression free interval of tumors ≤15 mm in diameter was 79.8% and that of tumors >15 mm in diameter was 28.6% (p = 0.001, log-rank test) (Figure 2).

**Figure 3:**
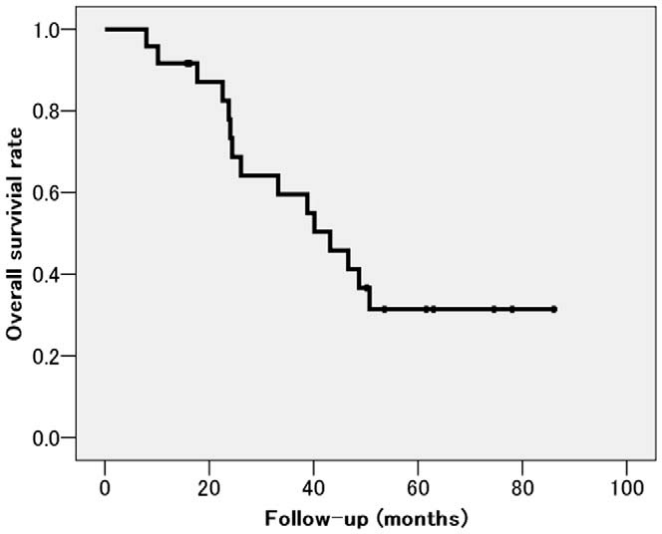
The overall survival curve after first cryoablation. Median overall survival was 43 months (8–86). One- and 3-year overall survival rates of 91% and 59.6%, respectively.

Median overall survival was 43 months (8–86). One- and 3-year overall survival rates of 91% and 59.6%, respectively. (Figure 3).

We also analyzed Local progression free interval, and overall survival according to age, and treatments before cryoablation. We compared the patients aged <63 versus > = 63, since 63 was the median age in this study group. When we divided the tumors as two groups by the age <63 (n = 29) versus > = 63 (n = 26), the progression free interval of the two groups has no significant difference by log-lank test (p = 0.77). Also, the survival of the two groups, <63 (n = 12) versus > = 63 (n = 12) did not differ significantly by log-lank test (p = 0.1). As for the treatment before cryoablation, when we divided the tumors into two groups with or without chemotherapy before cryoablation (n = 48 and n = 7, respectively), the local progression free interval of the two group did not differ significantly by log-lank test (p = 0.545). The overall survival of two groups (n = 17 and n = 7, respectively) also did not differ significantly by log-lank test (p = 0.568).When we divided the tumors in two groups with or without surgery before cryoablation (n = 27 and n = 28, respectively), the local progression free interval of the two group did not differ significantly by log-lank test (p = 0.220). The overall survival of two groups (n = 14 and n = 10, respectively) also did not differ significantly by log-lank test (p = 0.583). When we divided the tumors in two groups with or without radiosurgery before cryoablation (n = 5 and n = 50, respectively), the local progression free interval of the two group did not differ significantly by log-lank test (p = 0.158). The overall survival of two groups (n = 2 and n = 22, respectively) also did not differ significantly by log-lank test (p = 0.586).

## Discussion

Cryoablation has been performed for hepatic and for prostate cancer, and it has shown acceptable results; however, in most cases it was performed under general or epidural anesthesia. In our clinical study, all the procedures were performed under local anesthesia, which is similar to the conditions in the study by Wang et al. [14]. We therefore retrospectively evaluated the feasibility as well as the local control potential of cryoablation for lung metastasis from colorectal cancer.

With regard to the complications associated with cryoablation, the frequency of pneumothorax observed in our study was higher than the frequency of pneumothorax reported in a study by Wang et al [14]. In our study, we performed chest CT in all the cases one day after the procedure to check for the existence of pneumothorax. This may be why we found substantial number of asymptomatic pneumothorax. The results of our study were comparable with a report of percutaneous radiofrequency ablation by Yamagami T et al. [21], in which Grade 2 pneumothorax occurred in 19 of 129 sessions (14.7%), and the insertion of a chest tube was required in 5 sessions (3.9%).

There were some reports about radiofrequency ablation for lung metastases from colorectal cancer [22], [23]. In these reports, 1- and 3-year overall survival was 84–96%, 48–56%, respectively and 1- and 3-year progression free survival was 72–90%, 56–79%, respectively. These percentages in these studies were similar to our result.

There are several possible reasons for the local progression free interval difference depending on the tumor size: (1) The existence of a physical limitation in the ablation size when only 1 cryoprobe is used. Our experiments in pigs have shown that a cryoablation probe can freeze an area (known as an “ice ball”) of about 2 cm in diameter [17], [18]. (2) We still have little information on the size and shape of the ablation area when several probes are used simultaneously, although some experimental reports *in vivo* showed the thermal map of 2 probes [24] and the effect of freeze time [25]. (3) Patients with larger metastatic lung lesions have a greater probability of other metastatic disease including micro-metastases or satellite lesions [3], [26]. Improvement in local progression free interval, most probably by increasing the number of cryoprobes per tumor, will be necessary for future treatments.

One of the potential advantages of cryoablation is that cryoablation preserves the collagenous architecture and prevents the perforation and/or destruction of the cartilaginous rings of the bronchial tree [27]. Limited data are available about the effects on larger airways of cryoablation and of thermoablation. But case reports in the literature give the impression that radiofrequency ablation is more likely to result in larger air leaks that may progress to bronchopleural fistulae [28]. Moorjani et al reported that cryoanalgesia of −50°C for 1 minute on the intercostal nerves did not damage the neural structures. This allowed regrowth of the nerve through the perineurial canal [29]. This may be why intercostal nerves' pain in most patients in our study was recoverable, although most patients with thoracotomy suffer from irreversible pain. These features also suggest its superiority over heat-based ablation of lung tumors, especially near large vessels, the tracheobronchial region, or other mediastinal structures, though it has been reported to cause “cryoshock phenomenon” in hepatic cryoablation [30], [31], [32] and to cause renal parenchymal fracture in renal cryoablation [33].

The present study represents an early experience, with a small number of patients Hence extrapolation of the results to clinical practice should be performed with caution. Further confounding effects of the therapies received before cryoablation cannot be ruled out. Besides, the main limitation of this report is the lack of long-term follow-up data. Therefore, this was not a definitive study either for assessing the effects of cryoablation on patient survival, the effectiveness of cryoablation in the treatment of newly diagnosed lung metastasis from colon cancer, or for determining whether cryoablation was as effective as surgery for lung tumors.

In conclusion, percutaneous cryoablation may have a useful role in the management of colorectal pulmonary metastases less than 15 mm in diameter, when surgical resection is not an option. To realize the potential of this therapeutic modality for larger lesions, further research will be needed on the kinetics of heat transfer during freezing of pulmonary tumours.
